# Investigating Effects of Typographic Variables on Webpage Reading Through Eye Movements

**DOI:** 10.1038/s41598-019-49051-x

**Published:** 2019-09-03

**Authors:** Michele Scaltritti, Aliaksei Miniukovich, Paola Venuti, Remo Job, Antonella De Angeli, Simone Sulpizio

**Affiliations:** 1 Università degli Studi di Trento, Dipartimento di Psicologia e Scienze Cognitive, Rovereto, TN 38068 Italy; 2 Fondazione Marica De Vincenzi ONLUS, Rovereto, TN 38068 Italy; 3 Università degli Studi di Trento, Dipartimento di Ingegneria e Scienze dell’Informazione, Povo, TN 38123 Italy; 4 Libera Università di Bolzano, Facoltà di Scienze e Tecnologie Informatiche, Bolzano, BZ 39100 Italy; 5 Università Vita-Salute San Raffaele, Facoltà di Psicologia, Milano, 20132 Italy; 6 Università Vita-Salute San Raffaele, Centro di Neurolinguistica e Psicolinguistica, Milano, 20132 Italy

**Keywords:** Dyslexia, Reading

## Abstract

Webpage reading is ubiquitous in daily life. As Web technologies allow for a large variety of layouts and visual styles, the many formatting options may lead to poor design choices, including low readability. This research capitalizes on the existing readability guidelines for webpage design to outline several visuo-typographic variables and explore their effect on eye movements during webpage reading. Participants included children and adults, and for both groups typical readers and readers with dyslexia were considered. Actual webpages, rather than artificial ones, served as stimuli. This allowed to test multiple typographic variables in combination and in their typical ranges rather than in possibly unrealistic configurations. Several typographic variables displayed a significant effect on eye movements and reading performance. The effect was mostly homogeneous across the four groups, with a few exceptions. Beside supporting the notion that a few empirically-driven adjustments to the texts’ visual appearance can facilitate reading across different populations, the results also highlight the challenge of making digital texts accessible to readers with dyslexia. Theoretically, the results highlight the importance of low-level visual factors, corroborating the emphasis of recent psychological models on visual attention and crowding in reading.

## Introduction

Reading is a uniquely human ability and is strongly intertwined with human history. It exerts a pervasive influence on society, while also being shaped by its technological advancements. For example, printing press with movable types changed the physical layout of texts, while also enhancing their availability. In turn, the larger diffusion of reading material represented a turning point for modern societies. Nowadays, given the widespread use of personal computers, smartphones, and tablets, digital texts are gaining a prominent role in our daily life. More than 90% of young Europeans use computer and/or internet on a daily basis^1^. Thus, it is important to understand readability in the context of digital texts and, possibly, to enhance it for the largest number of users, including people with reading difficulties. In this article, we aim at shedding light on the factors that affect readability of webpages, taking into consideration different levels of reading experience (adults vs. children) and reading ability (typical readers vs. readers with dyslexia). We focus on the effects of webpage visuo-typographic characteristics – such as font type and size, spatial distribution of text, and the use of spacing – on eye movements during reading. There are both theoretical and empirical reasons to believe that these factors might lead to substantial increases in readability.

Texts represent complex, cluttered visual stimuli. An efficient visual processing of this sort of stimuli represents a crucial part in the development of reading abilities, and most likely entails a series of cognitive and neurobiological attunements to the specificities of the reading environment (e.g.^2^). According to recent theoretical proposals^3,4^, mechanisms of visuo-spatial attention play a pivotal role in overcoming the challenging features of orthographic decoding. For example, when readers are gazing a single word, they actually sweep a spotlight of attention serially across letters to aid their identification and the computation of the correct serial order^3^. Consistently, there seems to be a link between pre-reading abilities in orienting visual attention and future reading skills^5^; see also^6^. Indeed, impairments in orienting of spatial attention have been described as an important substrate of developmental dyslexia, a learning disability that affects 5–17% of the population^7^. Visual-typographic aspects of digital texts may facilitate the processing of orthographic input, reducing the demand on mechanisms of visual attention to support letters’ identification and the computation of their serial order^8^. Additionally, visuo-typographic variables may directly modulate crowding phenomena, where the identification of a visual target is made more difficult by the presence of distractor stimuli flanking the target itself^9–11^. Albeit ubiquitous in reading, crowding exerts an exaggerated impact in readers with dyslexia (e.g.^12,13^). Simple adjustments in the text’s visual format may significantly reduce this problem. For example, Zorzi and colleagues^14^ recently showed that increasing inter-letter spacing (together with space between words and interline space) significantly improves reading speed and accuracy for children with dyslexia (see also^15,16^; but see^17^).

Whereas from a psychological perspective the notion that typographic variables may significantly affect reading is mainly theory-driven, the field of human-computer interaction (HCI) considered these variables from the practical perspectives related to usability and accessibility (e.g.^18,19^). HCI has provided a number of readability guidelines, in order to summarize good practices in design and foster accessibility. Our previous work^20^ explored the relationships between user-rated readability and the compliance of texts with existing guidelines, using experts’ evaluation of compliance and users’ readability ratings collected online. Automatic measures were also developed for some guidelines targeting aspects such as size and types of font, luminance contrast, spacing, and spatial arrangement of texts (Table 2). These measures correlated with the perceived readability ratings (i.e., a subjective measure) provided by typical readers and readers with dyslexia. Albeit valuable to understand user experience, this approach did not tackle the link between typographic variables and actual reading processes, as the latter were measured indirectly, via user evaluation. Instead, in the present research we assessed the impact of typographic variables on measures of eye movements, in order to directly tackle the reading process in its unfolding.

Eye movements during reading are characterized by a sequence of jerky movements (saccades) followed by moments in which the eyes are held relatively still (fixations) on a target - typically a word - to allow its decoding and processing. In psycholinguistic research, eye movements proved to be sensitive to semantic (e.g.^21^), syntactic (e.g.^22^), lexical (e.g.^23^), and orthographic proprieties (e.g.^24^) of the stimuli, thus offering a window into the cognitive machinery underlying different levels of processing^25^. Eye movements also capture differences between readers, for example as a function of their age and reading experience^26–29^. Importantly, compared to typical readers, readers with dyslexia display an increase both in the number and in the duration of the fixations, whereas the amplitude of the saccades is reduced^25,30,31^. These differences are specifically related to language processing rather than oculomotor factors^30^, making eye movements a suitable tool to capture potential differences between typical readers and readers with dyslexia with respect to the influence exerted by visuo-typographic aspects.

Compared to the rich tradition of studies of reading focusing on cognitive and linguistic processes, studies of eye movements designed to assess typographic characteristics are underrepresented^32^. In their pioneering work, Tinker and collaborators (e.g.^33^) demonstrated that a number of typographic characteristics may exert an impact on eye movements during reading (for a review^34^). For example, reading speed decreases for texts using: (a) all capital letters, (b) very large or very small font sizes, (c) small line widths, (d) reduced luminance contrast between text and background, or (e) narrow interline spacing. More recently, other researchers both in the field of psychology^35^ and HCI^36–39^ explored the influence of typographic variables on eye movements.

Notably, these previous experiments used highly-controlled, ad-hoc stimuli and involved factorial manipulations of a limited number (or single) variables of interest. Albeit this approach has proven valuable, we argue in favor of a complementary alternative. Instead that directly manipulating the variables of interest within a limited set of artificial stimuli, we considered a relatively large sample of existing digital texts (webpages) retrieved from the web. This ecological perspective allows to jointly evaluate the effects of multiple co-occurring typographic characteristics featuring the real ranges of variability we typically encounter in daily situations. For example, most typographic measures vary along a continuum, and it is important to assess their effect by capitalizing on this feature, rather than by comparing artificially extreme configurations that might not represent what readers find in everyday materials. Additionally, different typographic variables tend to be related (e.g., font size and interline spacing) and it is thus important to jointly consider multiple predictors in order to assess their unique effect, over and above other co-varying features. These issues call for investigations relying on ecologically valid stimuli. Additionally, this provides the chance to test variables related to visual attention and crowding beyond the carefully controlled stimuli typically used in laboratory settings, potentially shedding light on their weight in everyday reading.

In our experiment, participants were asked to silently read texts within real webpages while their eye movements were recorded. A relatively large sample of webpages (N = 50) was used, to capture (part of) the natural variability of typographic factors across webpages. We assessed the impact of visuo-typographic variables on fixation durations, number of fixations, and amplitude of the saccades. Both young adults (university students) and children (middle-school students) were recruited. Both age groups included typical readers and readers with dyslexia, to address potential modulations of the effects of visuo-typographic factors as a function of age and reading ability.

## Methods

### Participants

A total of 85 Italian native-speakers were recruited from two age groups, adults (university students) and children (middle school students). Within each group, typical readers and readers with dyslexia were included. Albeit visual acuity was not assessed before the experiment – and this represents a limitation of the study as reduced acuity can affect eye movements parameters – all participants reported normal or corrected-to-normal visual acuity. None of them explicitly reported any visual difficulty during reading.

Four participants were excluded because they did not complete the experimental procedure, 1 for difficulties in tracking eye movements, and 1 because the reading scores were not compatible with the group he was recruited for (most of the reading scores were 2 SDs away from the normative mean, despite having been recruited for the typical reader group). The final sample included 79 participants.

Reading ability was assessed using a text reading task^40,41^, the Word Reading and the Nonword Reading subtests from the Developmental Dyslexia and Dysorthography Battery 2^42,43^. Time (syllables per second) and accuracy scores were considered, and raw-scores were converted to z-scores following normative data. Raven matrices were administered (Standard Progressive Matrices for the adults, Colored Progressive Matrices for children) in order to control for non-verbal reasoning abilities and intelligence across groups of participants. Intelligence quotient was estimated following normative data for the Italian population^44,45^. All the tests were either administered by the experimenter, or the corresponding scores were retrieved form the most recent evaluation performed by health services. Demographic, reading, and intelligence data are summarized in Table 1.

**Table 1:** Description of the participants. N = number of participants in the group; F = number of females in the group; SD = standard deviation; Years Edu = years of education; syl/s = syllables per second.

Measures	Adults		Children	
	Typical	With Dyslexia	Typical	With Dyslexia
N (F)	20 (15)	20 (9)	20 (10)	19 (7)
Age (SD)	23.55 (4.26)	22.43 (2.93)	11.55 (0.94)	11.47 (0.90)
Years Edu (SD)	16.45 (2.04)	16.05 (2.04)	5.95 (0.76)	5.79 (0.85)
Text syl/s (SD)	−0.11 (1.12)	−2.87 (1.85)	−0.32 (0.75)	−1.99 (0.62)
Text errors (SD)	0.50 (0.87)	4.21 (3.95)	—	—
Word syl/s (SD)	−0.16 (0.84)	−2.46 (1.01)	−0.40 (0.79)	−4.48 (4.66)
Word errors (SD)	−0.57 (0.46)	0.54 (1.55)	0.23 (0.68)	2.21 (1.88)
Nonword syl/s (SD)	−0.27 (0.99)	−2.30 (1.23)	−0.19 (0.92)	−2.09 (3.90)
Nonword errors (SD)	−0.33 (0.84)	0.95 (1.50)	−0.12 (1.06)	2.02 (1.94)
Raven IQ (SD)	121 (8)	118 (11)	110 (12)	108 (10)

For reading tests, we report z-scores calculated with reference to age-appropriate normative data. For children, z-score conversion for errors in text reading was not possible as normative data provide a categorical label for the performance. With respect to this index, within typical readers, only one child’s performance was labeled as below the norm. For children with dyslexia, 16 exhibited a performance labeled as below the norm. For 2 participants in the group of children with dyslexia, the local health service did not provide z-scores for errors in the word and nonword reading tasks. For one participant, both indexes were labeled as “within the normal range”, whereas for the other both were labeled as “below the 5^th^ percentile”. Error data for text reading were missing for one child of the readers with dyslexia.

All participants provided written informed consent prior to the experimental session. For minors, consent was obtained from their parents. The research has been conducted adhering to the ethical guidelines and the legal requirements of the country in which the study took place (Italy). The study was approved by the Ethic Committee of the University of Trento. Part of these data have been used in another study^46^ to assess and compare human-based and algorithm-based use of readability guidelines in webpage evaluation.

### Materials

We automatically sub-sampled 50 webpages out of an initial sample of 160 webpages, trying to maximize the combined variance across the predictor variables. The 50 pages were saved as screenshots (1600 pixels wide) and sliced in 1200-pixel high pieces if a page was too long to fit on the screen (mean N of pieces = 2.36, range 1–4, SD = 0.66). If the bottom slice was small and had no content, it was dropped. If it was shorter than 1200 pixels, the empty space was filled to keep the remaining webpage content at the top of screen and emulate the scrolling as it would happen naturally. These empty spaces were filled with dark-gray diagonal lines (Lum = 200 out of 255, 10 pixel thick, 45-degree inclination) on gray background (Lum = 150). The pages covered topics such as scientific/technological discoveries, cultural events, and health education. For each page, two simple comprehension questions were devised, with their corresponding alternative responses (one correct, one wrong). These questions were used during the experiment to ensure that participants actually read through the webpages.

Twenty pseudo-randomized lists of webpages were created for each group. Each participant saw 5 pages, so that each page was seen by two participants within each group.

### Apparatus and procedure

Participants sat 75 cm from a 21-in monitor (40 × 30 cm), with their head on a chin-rest. A keyboard was placed in front of them to navigate through the experimental procedure. Eye movements were recorded via a EYELINK 1000 PLUS system (desktop mount, 1000 Hz sampling rate; SR Research) from the right eye, except for 3 participants for which the left eye was tracked. Eye movements were recorded using parameters recommended for cognitive research, with saccadic detection based on a velocity threshold of 30°/s and an acceleration threshold of 8000°/s.

The experimental procedure was controlled by E-Prime 2 software. Written instructions were presented on the screen. For participants with dyslexia, the experimenter read them out aloud. Participants were instructed that the main task was to mentally read the text of the pages. They could explore the webpage before starting to read and/or when finished reading, but they were instructed to complete the reading of the text once they had begun. Participants were informed that 2 comprehension questions would follow each page. Five pages were presented to each participant. For pages extending over multiple screenshots, participants could reach the next portion of the page by pressing the down-key, and could return on the previous portion by pressing the up-key, without limitations. In this way we were able to exploit fine-grained monitoring and measuring of eye movements granted by the use of static stimuli, while also allowing the navigation of the content in a way that resembles the actual interaction with online material (e.g., pressing the spacebar in the browser results in a fast-scrolling similar to the one implemented here). Participants could switch off the page by pressing the space bar. Participants responded to the two 2-alternative forced-choice questions by pressing the number corresponding to the intended response (1 or 2) on the keyboard. Each question was presented with the two alternative responses and no time limit. Afterwards, participants were asked to rate, on a 1 to 7 scale, whether the topic was interesting or not (1- not interesting at all, 7- very interesting), how familiar they were with the website (1- never seen it, 7- very familiar), and who, in their opinion, was the target of the page (children, adults, both). Responses to an additional question regarding the perceived difficulty in reading the webpage were not recorded, due to a programming error. For readers with dyslexia, the experimenter read out aloud the questions after the participants finished reading.

After each page, participants could take self-terminated breaks. A calibration procedure took place before each new page. Participants were required to fixate for 1 s a small circle appearing on the screen. The circle appeared in 9 different locations of the screen (top, middle, and bottom portions of the left, central, and right part of the screen), in random sequence, except for the first and the final circles that appeared at the center of the screen.

Before the experimental phase, participants performed the Raven Matrices test. Reading tests were administered after the experimental phase. For most participants in the groups of readers with dyslexia, results of these tests were already available, in total or in part, from evaluations conducted by health services. In such cases, we took the scores from the last available evaluation, without re-administering the tests.

### Predictor variables

#### Linguistic predictors

We considered two psycholinguistic variables: word frequency, and word length. Word frequency values (log-transformed) were retrieved from the SUBTLEX-IT database^47^ for each word and then averaged within each page. Word length was measured as the number of characters for each word, and then averaged within each page.

#### Visuo-typographic predictors

On the basis of a review of the available guidelines for webpage readability in dyslexia, we identified 11 visuo-typographic variables deemed to affect reading and amenable to be measured via algorithms^20^. These measures, and information about how they were computed, are summarized in Table 2. Correlations between all the measures used as predictors are reported in the Supplementary Information (Table S1).

**Table 2:** Visuo-typographic predictors.

Predictors	Description
Amount of text	Combined length of all page texts, after empty-space and invisible characters are removed.
Page length	The length of page in pixels
Luminance contrast	Text-background luminance contrast L = (L_lighter_ + 0.05)/(L_darker_ + 0.05), with L_lighter_ and L_darker_ being the lighter and darker of the text and background luminance pair normalized to the 0 to 1 interval. Contrast estimates were weighted by the length of of the corresponding text, summarized, and normalized by the length of all page texts
Font size	Number of text blocks for each font size, weighed by font size, and normalized by the number of all text blocks.
Line spacing	For each piece of text, height of text piece minus the height of the text itself, normalized by the height of the text. The number of characters in each piece of text was weighted by the calculated index
Font type	Proportion of text styled in sans serif fonts
Bold	Proportion of bold text
Italic	Proportion of italic text
Underlined	Proportion of underlined text
Headers	Proportion of text in headers and titles (relative to all text)
Column width	For each text block, we calculated the maximum number of characters that fit in a line without overflowing on the next line. The estimates were weighed by the length of text in the blocks, summarized across blocks, and normalized by the number of all page characters
Left alignment	Ratio of left-aligned text to all text

For Luminance Contrast, cf. the WCAG 2.1 formulae https://www.w3.org/TR/WCAG21/#dfn-contrast-ratio.

### Measures of eye movements

When examining global indexes of eye movements in reading, reliance on a single measure can be misleading. For example, a decrease in average fixation duration for a text could be offset by an increased number of fixations^32^. The total number of fixations, on the other hand, might be a byproduct of perceptual span, but is also influenced by regressions and re-reading. It is thus important to consider multiple indexes. Here, we relied on (1) average fixation duration, (2) number of fixations, and (3) average amplitude of the saccades. Average fixation duration is assumed to capture the time, and thus the effort, needed to decode and process the orthographic input. Longer durations are assumed to signal less readable texts. For texts, number of fixations and average amplitude of the saccades map onto the construct of perceptual span (e.g.^25,32^), that is the amount of information that can be processed during fixation. When this amount decreases, the saccades will cover a smaller extension, and more fixations would thus become necessary to finish reading. Further, in our work, number of fixations captures also the potential need of a second reading for some part of the text. Increased number of fixations and reduced saccade amplitude would thus be both associated to reduced readability.

To extract these three measures, for each webpage all the parts of the main text were identified as areas of interest (AIs). This segmentation excluded pictures and any other element that was not part of the main text (e.g., links, advertisements). Eye movement analysis was limited to the phenomena detected within the AIs, in order to better capture eye movements related to actual reading.

#### Average fixation duration

Within each page, blink-related artifacts were removed, by excluding fixations recorded immediately before or after the blinks and lasting less than 100 ms. For each participant and each page, durations of all the fixations (within the text area) were averaged together, obtaining average fixation duration. This measure was used as an index of the effort needed to decode and process the orthographic input provided by the texts.

#### Number of fixations

The same fixations used to compute average fixation durations were used for this measure. The total number of fixations was counted within each webpage and each participant. This measure captures both perceptual span (together with the average amplitude of the saccades, described below), as well as regressions or second-pass reading.

#### Average amplitude of the saccades

For each page, saccades containing blinks were removed. Further, only saccades having their starting and end points within the textual AIs were retained, in order to focus on reading related movements. The amplitudes of these selected saccades were averaged within each webpage and within each participant. Together with number of fixations, this measure was mainly considered as an index of perceptual span.

### Data analyses

Fixation durations and amplitudes of the saccades were log-transformed prior to averaging, in order to better approximate a normal distribution.

Analyses were conducted using linear mixed-effects models to assess the unique impact of the predictors on our dependent variables. Both participants and webpages were considered as random factors, and modeled as random intercepts. All continuous predictors were centered. Analyses were performed in R^48^ using the package lme4, version 1.1–14^49^. Figures were made using the package ggplot2^50^.

For all the dependent variables, we first fitted models including the factors Age (adults vs children), Reading Ability (typical readers vs readers with dyslexia), and their potential interaction. We then considered linguistic predictors, and potential interactions with Age or Reading Ability. Before moving to proper visuo-typographic variables, we also added measures related to the length of text (Text Amount), the size of the pages (Page Length), and their potential interactions with Age and Reading Ability, to partial out the differences related to texts of different size. Following these preliminary steps, the visuo-typographic predictors were considered in 4 sequential blocks. In a first block, we assessed the impact of Luminance Contrast. In the second, we considered Font Size and Line Spacing. In the third block we considered the predictors Font Type, Bold, Italics, Underlined, and Headers. In the fourth block we assessed the impact of Column Width and of the proportion of Left-Aligned text. The reasoning behind this sequential block-wise assessment was to address first low-level visual factors related to contrast (block 1), crowding (block 2), and then progressively move towards higher level factors such as font specificities (block 3) and spatial organization of the paragraphs/texts (block 4). All the variables within each block were inserted together, in order to exclude the variance shared by multiple predictors from the assessment of the resulting model^51^.

Blocks of predictors were statistically assessed via model comparisons. Specifically, for each block, we first included the predictors in additive terms, and then also tested their interactions with Age and Reading Ability. Interactions were retained only if a log-likelihood test resulted in a significant increase of explained variance with respect to the model with additive terms. If the test failed, effects in additive terms were retained only if they resulted in a significant increase of explained variance with respect to the last model fitted before entering the new block of predictors. Before moving to each new block of predictors, we ran chi-square log-likelihood tests in which each single fixed-effect was assessed by comparing the full model against simpler models in which the fixed-effect under examination was excluded. The model was refitted excluding those terms that failed to highlight a significant contribution (stepwise backward elimination). This procedure was conducted in compliance with the principle of marginality, starting with higher-order interactions and moving backward, in case of non-significance, to the evaluation of the lower-order terms. In case the higher-order term was significant, we retained all the lower-order terms (i.e., if a three-way interaction was significant, we stopped the stepwise backward evaluation for the predictor involved and retained all the two-ways interactions and the main effect). The simplified model obtained via stepwise backward elimination was tested (a) against its counterpart with all the predictors, to make sure it did not lose a significant portion of explained variance, and (b) against the last model fitted for the previous block of predictors, to confirm the increase in explained variance.

## Results

Model comparisons for all the three measures of eye movements are listed in the Supplementary Information (Table S2).

### Average fixation duration

**Figure 1:**
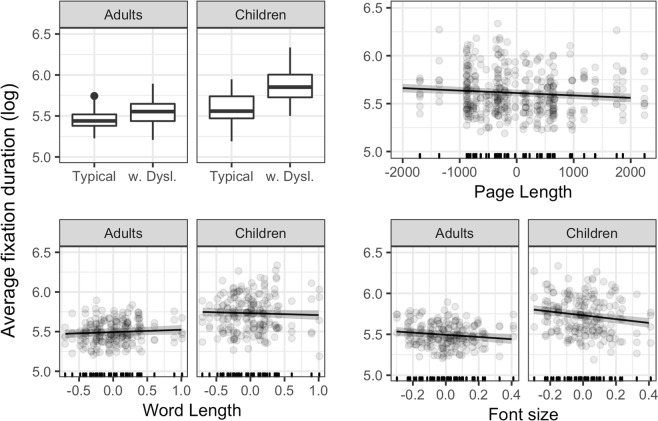
Significant effects for average fixation durations. Lines represent the estimated effects, and the shaded area represents 95% confidence intervals. w. Dysl. = readers with dyslexia.

Significant effects are represented in Fig. 1. For average fixation duration, we found a significant interaction between Age and Reading Ability. Fixations were longer for participants with dyslexia, and the difference with typical readers was larger in children than in adults. There was a main effect of Page length, with shorter fixations for longer pages. Word Length interacted with Age: whereas for adults fixations tended to last longer in texts with longer words, for children –surprisingly– the opposite was true. Finally, there was a main effect of Font Size, as fixation duration decreases with increasing font size. The effect is larger in younger readers. Table 3 reports the parameters of the final model.

**Table 3:** Parameters of the models.

Fixed Effects	b	SE	t
***1. Average Fixation Duration***			
Intercept	5.48	0.03	160.46
Age Group (Children)	0.15	0.05	3.18
Reading Ability (Readers with dyslexia)	0.09	0.05	2.01
Word Length	0.03	0.02	1.59
Page Length	−2.54e-05	7.31e-06	−3.48
Font Size	−0.13	0.04	−3.12
Age Group (Children) * Reading Ability (Readers with dyslexia)	0.18	0.07	2.63
Age Group (Children) * Word Length	−0.05	0.01	−3.87
Age Group (Children) * Font Size	−0.10	0.03	−3.09
***2. Number of Fixations***			
Intercept	5.78	0.07	77.29
Age Group (Children)	0.18	0.08	2.37
Reading Ability (Readers with dyslexia)	0.32	0.08	4.22
Amount of Text	2.97e-04	3.63e-05	8.17
Page Length	−4.12e-05	7.02e-05	−0.59
Headers	−0.92	0.40	−2.32
Column Width	0.03	0.10	0.37
Left Align.	−0.26	0.12	−2.09
Age Group (Children) * Amount of Text	−5.95e-05	2.15e-05	−2.77
Age Group (Children) * Page Length	1.14e-04	4.20e-05	2.71
Age Group (Children) * Column Width	−0.14	0.08	−1.78
Reading Ability (Readers with dyslexia) * Column Width	−0.12	0.07	−1.96
Age Group (Children) * Reading Ability (Readers with dyslexia) * Column Width	0.33	0.10	3.24
***3. Average Saccade Amplitude***			
Intercept	0.30	0.04	7.33
Age Group (Children)	−0.27	0.04	−5.73
Reading Ability (Readers with dyslexia)	−0.21	0.05	−4.60
Amount of Text	1.62e-05	8.52e-06	1.90
Font Size	0.43	0.08	5.24
Line Spacing	0.52	0.24	2.18
Age Group (Children) * Amount of Text	1.30e-05	5.92e-06	2.20
Age Group (Children) * Line Spacing	−0.42	0.17	−2.55

Age Group (Adults vs Children); Reading Ability (Typical Readers vs Readers with dyslexia); the two variables were dummy coded using Typical Adults as reference. SE = standard error.

### Number of fixations

**Figure 2:**
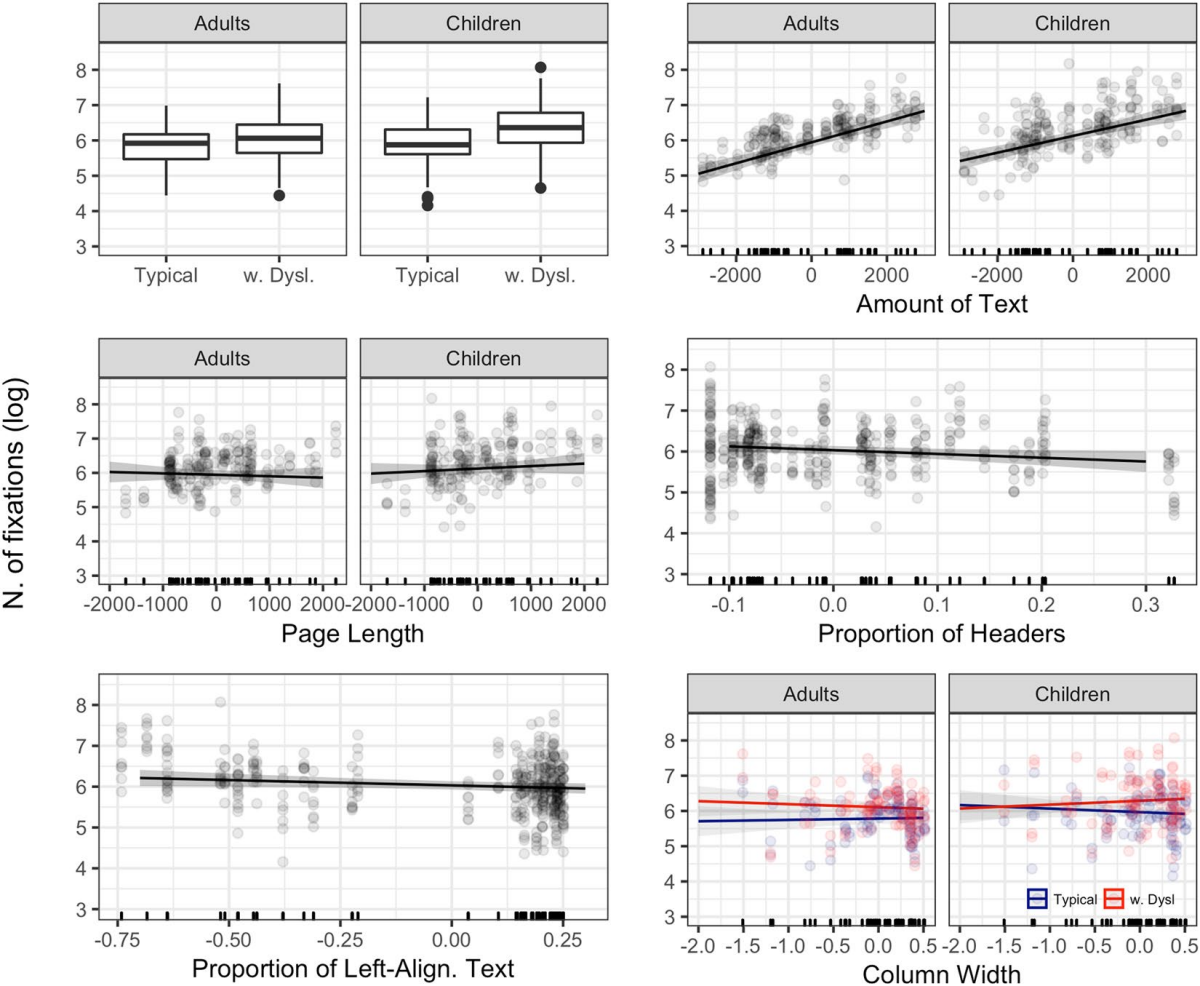
Significant effects for number of fixations. Lines represent the estimated effects, and the shaded area represents 95% confidence intervals. w. Dysl. = readers with dyslexia.

Significant effects are represented in Fig. 2. Age and Reading Ability yielded additive effects, as the difference between typical readers and readers with dyslexia was similar across adults and children, with participants with dyslexia performing more fixations in both groups. Number of fixations increased with increasing Amount of Text and, surprisingly, this pattern appeared to be mitigated in children. Also, whereas adults tended to make fewer fixations with increasing Page Length, the opposite pattern was found in children. The effects of Proportion of Headers and Proportion of Left-aligned text revealed that readers tended to make fewer fixations while reading left-aligned texts and texts using more headers, with no major differences across groups. Finally, we found a three-way interaction between Age Group, Language Group, and Column Width, seemingly driven by the fact that whereas for adult with dyslexia and typically reading children the number of fixations tends to decrease for wider texts, the opposite is true for children with dyslexia. The parameters of the final model are reported in Table 3.

### Average amplitude of the saccades

**Figure 3:**
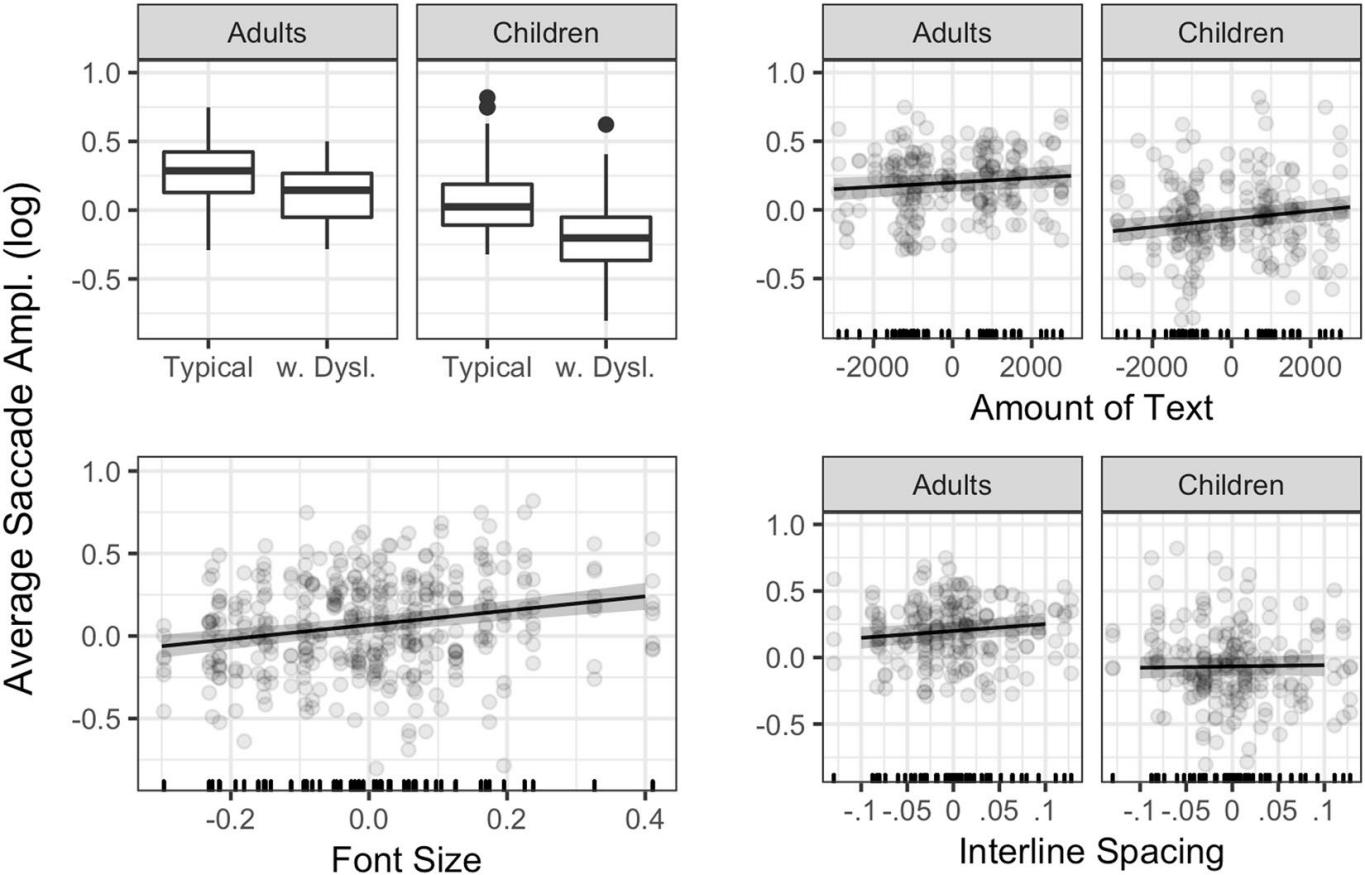
Significant effects for average saccade amplitude. Lines represent the estimated effects, and the shaded area represents 95% confidence intervals. w. Dysl. = readers with dyslexia.

Significant effects are represented in Fig. 3.

In terms of average amplitude of the saccades, Age and Reading Ability yielded additive effects. Saccades were smaller for children compared to adults, and for readers with dyslexia compared to typical readers. Clearly, the amplitude of the saccades increases with bigger font size. Further, we detected a significant interaction between Age Group and Amount of Text. Both adults and children made larger saccades in longer texts, but the pattern is emphasized in children. Finally, there was a significant interaction between Age and Interline Spacing. Whereas adults seem to benefit from larger interline space, which yields larger saccades, for children this visuo-typographic parameter has no effect. The parameters of the final model are reported in Table 3.

## Discussion

Multiple typographic variables (Table 4) exhibited significant effects on Webpage reading as indexed by eye movements. Importantly, we report effects detected when jointly considering multiple visuo-typographic factors in their natural range of variability within ecologically valid materials, rather than using artificial stimuli where extreme manipulations of single factors can result in unrealistic stimuli, thus limiting the scope of generalizations. Most of these effects were homogeneous across different groups of readers, or mainly sensitive to the readers’ age. In the reminder of the discussion we will tackle these different effects and discuss them with reference to both the theories of reading and the guidelines devised to enhance the accessibility of digital texts.

**Table 4:** Guidelines for text readability and empirical evidence from the present study.

Guidelines	Empirical support
Limit the amount of content on a page	yes
Ensure high luminance contrast between text and background,	null effect
Use a minimum of text size 12 pt or 14 pt	yes
Keep the between-line spacing of 1.5 point	yes
Avoid using italics	null effect
Use bolding to highlight	null effect
Avoid underlining large blocks of text	null effect
Use a plain, evenly spaced sans serif font	null effect
Use section headings	yes
Avoid formatting texts in large-width columns	partial
Use left-justified text with ragged right edge.	yes

All the indexes of eye movements we considered (average fixation duration, number of fixations, and amplitude of the saccades) exhibited clear differences among age groups as well as between typical readers and readers with dyslexia. Adults displayed shorter and fewer fixations, and larger saccades than children, and typical readers displayed shorter and fewer fixations, and larger saccades than readers with dyslexia. These results replicate those of the extant literature^25,30,31^. This is important, as our methodology represents a departure from the traditional psycholinguistic experiments in which these differences were originally reported. Only average fixation duration revealed the presence of an interaction between Age Group and Reading Ability, indicating that the difference between typical readers and readers with dyslexia was larger in children than in adults. Since fixation durations mainly reflect (among other things) the ease of orthographical and lexical processing^25^, reading experience and ability seem to yield multiplicative effects on these processes.

Before addressing fine-grained typographic variables, our analyses considered predictors related to the amount of text participants had to read and to the size of the webpages. Text Amount yielded reliable effects, with longer texts yielding more fixations and larger saccades. Interestingly, for children the increase in number of fixations with longer texts was attenuated, whereas the enhanced amplitude of the saccades for longer texts was exaggerated. This may suggest that under more demanding conditions (i.e., larger amount of text to be read), children may have attempted to reduce the effort by skimming through text, thereby reducing the number of fixations and increasing the amplitude of saccades. In contrast, the size of the page (Page Length) affected all groups of readers, reducing the average fixation duration in longer pages. This was true even when the effect of Text Amount was controlled for, suggesting that webpage size influences reading over and above the amount of text. This result points toward a facilitation for less cluttered reading materials. Longer pages would reduce crowding by increasing the ratio between empty space and content. All other aspects being equal, a larger page would diminish visual interference by allowing more spacing between different visual elements.

Luminance Contrast failed to display any sizeable effect on measures of eye movements, suggesting that when considered in its natural range of values rather than using extreme comparisons (e.g.^33^), this variable exerts a limited influence on reading. Differently, typographic measures related to crowding phenomena, such as Font Size and Line Spacing, yielded reliable effects. Increasing font size reduced average fixation duration, replicating results from factorial manipulations^38^. The effect is stronger in younger readers, but does not differ between typical readers and readers with dyslexia. This latter finding appears to be counterintuitive, considering that font size is closely related to crowding phenomena and that readers with dyslexia are particularly sensitive to them. Our materials may not have included fonts large enough to detect the enhanced benefit for readers with dyslexia reported in previous research, which used font sizes larger than those featured in typical webpages (e.g.^39^). In fact, albeit our texts included font size ranging from 5 to 53 points (in log-values, 1.61–3.97), the aggregated average font size per page ranged from 10 to 18.46 points. Yet, increasing font size still produced a significant reduction in fixation times for all readers. When considering adult participants, the estimates of our model (Table 3) show a difference of ~20 ms in average fixation duration between the text with the smallest font size and the text with the largest one. A typical webpage requires hundreds of fixations and a 20 ms reduction per fixation adds up to a substantial decrease in terms of overall reading time. Larger font size also increases the amplitude of saccades, without influencing the number of fixations. Thus, rather than modulating the perceptual span, larger font size might simply require slightly larger saccades to accommodate the increased size of the orthographic stimuli.

Line Spacing selectively modulates the amplitude of saccades, with increased amplitudes at increasing spacing. In our sample of stimuli, Line Spacing ranged from 40% to 350% of the corresponding text font size (from 110% to 201% when considering values averaged within each page). One possibility is that the saccade amplitude is enhanced simply because with increased interline spacing the eyes need to travel a longer distance to reach a new line. If this had been the case, no difference as a function of age would have been expected, whereas we observed the pattern to be enhanced in adult readers. We speculate that the effect is related to the reduction of crowding effects when line spacing increases^52^. As crowding phenomena are reduced for vertically arranged stimuli^53^, only more experienced readers might take advantage of a reduction in “vertical” crowding, given its secondary influence compared to the influence triggered by flanking stimuli. However, under these circumstances, one would expect the effect to be different for adults with and without dyslexia – a pattern that we did not observe. Our interpretation of the effect of Line Space thus warrants some caution. The manipulation of line spacing has indeed yielded inconsistent findings across the literature, including null, beneficial and detrimental effects (see^36,39^).

Font-style predictors (bold, italic, underline, serif) did not impact the reading measures even though extant guidelines provide explicit recommendations regarding these features^54,55^. However, in line with the guidelines^54,56^, the use of headers produces a benefit for all groups of readers by reducing the number of fixations. In our data, the number of fixations is determined not just by the readers’ visual span, but also by regressions and second-pass reading. As the presence of headers did not concurrently affect the amplitude of the saccades, the effect might indicate a reduced need for second-pass reading of the textual material when headers are used.

Finally, variables related to the spatial disposition of the texts within the webpages highlighted a few interesting effects. The use of Left-Aligned Text facilitates reading by reducing the number of fixations, possibly because such format prevents the “river effects” generated by the coincidental vertical alignment of spaces^57^. Again, this corroborates the recommendations from the guidelines^54,55^. The effect of Column Width, on the other hand, reveals a more complex pattern. Extant evidence suggests that narrower columns yield fewer fixations in typical adult readers^37^ as well as in high-school readers with dyslexia^35^. For the former group, the effect has been linked to a reduction in regressions, and to the fact that return sweeps (when the eyes move from the end of a line onto the beginning of the next one) become more difficult in larger texts, often requiring corrections via additional fixations due to the greater distance the eyes need to travel. For high-school readers with dyslexia, the effect has been linked to mechanisms of visual attention and crowding. Specifically, narrower column-widths would minimize the interference exerted by stimuli at the left of fixations, which exert an exaggerated influence in readers with dyslexia, possibly due to sluggish visual attention^35^. The results we obtained for typical adult readers and for children with dyslexia corroborate these past findings. For typical adult readers, our model estimates a ~10% increase in number of fixations when moving from the narrowest-column texts to the widest-column (301 vs 331). Readers with dyslexia in the children group displayed a similar pattern, with a ~18% increase (482 vs 569). In contrast, both adults with dyslexia and typical children exhibited the opposite pattern, with – respectively – a ~19% (532 vs 430) and a ~22% (476 vs 370) decrease in estimated number of fixations when moving from narrower to wider formatting. We speculate that, in these latter populations, the benefits related to narrower texts (easier return sweeps and reduced interference from stimuli at the left of fixations) were not strong enough to counteract the disruptive consequences of the more frequent return sweeps. Clearly, this pattern of results calls for additional investigations.

In summary, our results show that several visuo-typographic features captured in their natural variance exert an impact on reading processes as indexed by eye movements. Effects stemming from lower-level variables, such as font size, page size and line spacing seem to support the importance of crowding phenomena and visual attention in shaping the reading behavior. Notably, our results demonstrate these effects using ecologically valid stimuli both in terms of content^58^ and visual appearance. This corroborates the most recent models of (eye movements in) reading, in which the aim is to provide an overarching framework encompassing the whole chain of processes from the earliest stage of visual word recognition (letter identification) onto text reading. In one of the most recent computational instantiation of such models^4^, crowding and visual attention are implemented as specific parameters weighting the visual input. Our results further support the importance of these factors in everyday reading and the need to explicitly consider them within reading models.

Other than theoretically contributing to the understanding of how surface features of (web)pages may affect reading processes, our results also provide useful information in order to enhance readability of web texts by providing experimental support for several Web design guidelines (Table 4). Interestingly, many variables seem to yield comparable benefits across all of the examined groups of readers. Whereas most of the existing Web readability guidelines focused either on dyslexia (e.g.^54,55^) or typical readers^19^, researchers have recently advocated the need for inclusive recommendations that may benefit all users^59^. Our results support this approach and highlight several simple recommendations for webpage design that would facilitate reading for typical readers and readers with dyslexia alike. At the same time, it must be stressed that none of our results suggests that visuo-typographic variables may fully compensate for the difficulties faced by readers with dyslexia. We maintain that a careful consideration of these variables may reduce effort and fatigue for readers with dyslexia and typical readers, without any cost for either of the groups. Yet, further improvements for readers with dyslexia will likely stem from more extreme manipulations of typographic aspects (e.g.^14,39^) compared to what is found in the natural variety of standard (web)pages targeting the general audience. Future research will need to evaluate whether these more extreme formats can be implemented in standard reading materials, given the other constraints (e.g., size and number of pages needed to accommodate these layouts). Further, any potential benefit would have to be evaluated against the fact that such formats might not be suited for typical readers^17,60^.

Extant guidelines suggest that several typographic variables may impact reading behavior. Sometimes, recommendations about how to handle these factors are controversial^20^. Here we offer empirically and experimentally grounded indications about the variables that play a major role and the direction of the corresponding effects. Albeit some of our results can be already transferred into practice, they may also provide indications for further investigations, where variables highlighted here (Table 4) can be more thoroughly investigated to find the boundary conditions of their effects and their influence across different population of readers. We argue that these potential future developments would be intriguing (a) theoretically, to approach a naturalistic study of reading, (b) methodologically, to bridge the gap between psycholinguistics and HCI research, and (c) practically, in order to enhance the accessibility of information contained within digital texts.

## Supplementary information

Supplementary Information

## Data Availability

The datasets generated and/or analysed during the current study are not publicly available because we did not obtain consent for publication from the participants. Data are available from the corresponding author on reasonable request.
