# Supplementary material for: Investigating Effects of Typographic Variables on Webpage Reading Through Eye Movements
Source: Sci Rep. 2019 Sep 3;9:12711. doi: 10.1038/s41598-019-49051-x (PMC6722069; doi:10.1038/s41598-019-49051-x)
Supplement: Supplementary file 1 — Supplementary Information [file 41598_2019_49051_MOESM1_ESM.docx]

Investigating Effects of Typographic Variables on Webpage Reading Through Eye Movements

Michele Scaltritti, Aliaksei Miniukovich, Paola Venuti, Remo Job, Antonella De Angeli, and Simone Sulpizio

**Supplementary Information**

|  | 1 | 2 | 3 | 4 | 5 | 6 | 7 | 8 | 9 | 10 | 11 | 12 | 13 | 14 |
| --- | --- | --- | --- | --- | --- | --- | --- | --- | --- | --- | --- | --- | --- | --- |
| 1 Freq. | - |  |  |  |  |  |  |  |  |  |  |  |  |  |
| 2 W. Length | -.81 | - |  |  |  |  |  |  |  |  |  |  |  |  |
| 3 Text am. | .01 | -.13 | - |  |  |  |  |  |  |  |  |  |  |  |
| 4 P. Length | .01 | -.16 | .64 | - |  |  |  |  |  |  |  |  |  |  |
| 5 Lumin. | -.02 | .06 | -.21 | -.29 | - |  |  |  |  |  |  |  |  |  |
| 6 Font size | -.04 | -.07 | .04 | .25 | -.12 | - |  |  |  |  |  |  |  |  |
| 7 Line spacing | .02 | .02 | -.15 | -.05 | -.05 | -.24 | - |  |  |  |  |  |  |  |
| 8 Font type | -.12 | .12 | **-.34** | **-.31** | .08 | **-.31** | .03 | - |  |  |  |  |  |  |
| 9 Italic | -.11 | .09 | **.35** | .12 | -.04 | .01 | .09 | -.25 | - |  |  |  |  |  |
| 10 Underlined | .12 | .01 | .03 | -.15 | -.07 | **-.31** | **.37** | .23 | .13 | - |  |  |  |  |
| 11 Bold | -.17 | .10 | -.04 | .20 | -.19 | **-.32** | .15 | **.35** | -.13 | .06 | - |  |  |  |
| 12 Headers | .12 | -.16 | .14 | **.55** | **-.30** | **.29** | .15 | -.11 | .10 | -.11 | .25 | - |  |  |
| 13 C. width | .13 | -.08 | .01 | **.33** | -.13 | -.04 | -.09 | -.03 | -.02 | -.04 | .26 | **.47** | - |  |
| 14 Left align. | .21 | -.16 | -.13 | .04 | .07 | .08 | .09 | **-.28** | **-.28** | -.08 | -.17 | **.28** | .12 | - |

**Supplementary Table S1.** Correlations between predictors (Pearson’s r). Significant correlations are in bold.

| Block | Fixed Effects | Fixation duration | N. of fixations | Sac. amplitude |
| --- | --- | --- | --- | --- |
| - | Age + Reading | χ^2^ (2) = 52.60*** | χ^2^ (2) = 20.74*** | χ^2^ (2) = 41.75*** |
|  | Age*Reading | χ^2^ (1) = 6.86** | χ^2^ (1) = 1.59 | χ^2^ (1) = 1.94 |
| - | Freq. + Length | χ^2^ (2) = 3.68 | χ^2^ (1) = 5.22* | χ^2^ (2) = 3.79 |
|  | Freq*Age*Reading + Length*Age*Reading | χ^2^ (6) = 18.73** | χ^2^ (6) = 2.96 | χ^2^ (6) = 7.03 |
|  | Text am. + Page length | χ^2^ (2) = 11.89** | χ^2^ (2) = 51.20*** | χ^2^ (1) = 4.88* |
|  | Text am*Age*Reading + Page length*Age*Reading | χ^2^ (6) = 3.10 | χ^2^ (6) = 14.09* | χ^2^ (6) = 11.25° |
| 1 | Lumin | χ^2^ (1) = 0.70 | χ^2^ (1) = 1.55 | χ^2^ (1) = 1.61 |
|  | Lumin* Age*Reading | χ^2^ (3) = 3.53 | χ^2^ (3) = 0.61 | χ^2^ (3) = 0.13 |
| 2 | Font size + Line space | χ^2^ (2) = 19.06*** | χ^2^ (2) = 1.46 | χ^2^ (2) = 21.83** |
|  | Font size*Age*Reading + Line space*Age* | χ^2^ (6) = 14.57* | χ^2^ (6) = 1.30 | χ^2^ (6) = 12.86* |
| 3 | Font type + Bold + Italic + Underlined + Headers | χ^2^ (5) = 8.32 | χ^2^ (5) = 12.85* | χ^2^ (5) = 5.77 |
|  | Font type*Age*Reading + Bold*Age*Reading + Italic*Age*Reading + Underlined*Age*Reading + Headers*Age*Reading | χ^2^ (15) = 10.71 | χ^2^ (15) = 16.6 | χ^2^ (15) = 6.08 |
| 4 | Column width + Left alignment | χ^2^ (2) = 2.08 | χ^2^ (2) = 5.36° | χ^2^ (2) = 1.35 |
|  | Column width *Age*Reading + Left alignment*Age*Reading | χ^2^ (6) = 7.87 | χ^2^ (6) = 13.48* | χ^2^ (6) = 5.15 |

**Supplementary Table S2.** Model comparisons. Age = Age Group (Adults vs Children); Reading = Reading Ability (typical readers vs readers with dyslexia dyslexic); Freq. = Frequency; Lumin = luminance contrast; Text am = Amount of Text. Fixed effects were added incrementally following the order reported in the table. Additive models were tested against the last model that featured the inclusion of a new predictor. Models with interactions were tested against their additive counterpart. Before moving to the next block of predictors, fixed effects were assessed via stepwise backward elimination and only significant ones were retained (see text for details). *** = p < .001; ** = p < .01; * = p < .05; ° = p < .01
